# Linking Rheology and Printability for Dense and Strong Ceramics by Direct Ink Writing

**DOI:** 10.1038/s41598-017-06115-0

**Published:** 2017-07-20

**Authors:** Amin M’Barki, Lydéric Bocquet, Adam Stevenson

**Affiliations:** 1 Saint-Gobain CREE/Université Lyon I, LSFC Lab, Cavaillon, 84300 France; 2 Ecole Normale Supérieure, LPS Lab, Paris, 75005 France; 3 Saint-Gobain CREE, LSFC Lab, Cavaillon, 84300 France

## Abstract

Direct ink writing of dense and strong ceramic objects remains an important open challenge. We develop a universal dimensionless criterion for printing such objects. Boehmite, an Al_2_O_3_ precursor, was used to assess the rheological properties leading to dense structures in ceramics manufactured by direct ink writing. Boehmite suspensions undergo time dependent gelation, thus providing a rheological laboratory of flow behaviours that can be correlated with printability requirements. We measured the evolution of rheological properties over several days and quantified the deformation of simple printed shapes at different aging times. We then identified the relevant physical parameters leading to printable suspensions. We defined a dimensionless number, Ξ, based on measured rheological properties, that predicts deformation of the printed object and determines the printability criterion. An important difference with this criterion is that Ξ necessarily accounts for capillary forces and gravitational slumping. We show that boehmite inks reach a printed shape fidelity > 90% when Ξ > 1, and that Al_2_O_3_ bars printed under these conditions can be sintered to 97% density, without printing defects, and have flexural strengths (500–600 MPa) competitive with commercial aluminas. Using Ξ, researchers can rationally design inks for printing dense materials by tailoring their rheological properties such that Ξ ≈ 1.

## Introduction

A major goal of ceramic processing science is to produce complex three dimensional structures with excellent mechanical and/or functional properties. Multiple technologies where green bodies are built up layer-by-layer from simple building blocks have been developed to extend the range of structures available in ceramic materials. These processes are known by many names including additive manufacturing, 3D printing, and solid freeform fabrication. One additive manufacturing technique that is of particular interest due to its ability to produce space filling and spanning structures and relatively easy access to multimaterial printing is direct ink writing (DIW).

During direct ink writing, a colloidal suspension is passed through a computer controlled needle depositing a spatially controlled continuous filament of material. This process imposes stringent requirements on the rheological behaviour of the inks; they must easily flow through a narrow opening, but they must also resist deformation immediately after printing.

There are two key strategies for tailoring this type of rheological behaviour. One approach is to produce a low viscosity ink that undergoes gelation after printing so that it rapidly develops a high enough yield strength, through gelation, to resist deformation after printing. A second approach is to produce inks with precisely controlled rheological properties. Suspensions must be viscoelastic, with a sufficient yield stress to support layer stacking. It is also essential to obtain a shear-thinning flow behaviour to facilitate deposition through the needle. These rheological properties are often linked and cannot, in general, be varied separately. In the present article, we will focus on the second approach.

The rheological criteria for direct ink writing of spanning structures has been thoroughly evaluated, and many different spanning structures have been developed using a wide variety of colloidal and sol-gel inks^1–3^. However, there have been relatively few reports of dense, space filling structures produced by direct ink writing. The key factor in dense structures is that the cylindrical depositions from the nozzles must flow enough after deposition to fill the inevitable void space formed when stacking cylinders, and must then resist larger scale slumping of the printed object. Achieving this narrow range of rheological behaviour has been elusive. There are however a few exceptions^4, 5^ were dense samples were printed by DIW, but no rheological printability criterion was established.

In this work, we define the rheological properties that lead to strong and dense materials by direct ink writing.

We chose to develop robocasting inks based on boehmite gels, a well-known and industrially important *α*- Al_2_O_3_ precursor that results in highly dense, fine grained materials when processed appropriately. Boehmite gelation occurs by the partial dissolution of boehmite particle surfaces^6, 7^ into monomeric aluminium species Al(H_2_O)_6_^3+^, through successive hydrolysis and condensation reactions^8, 9^. Most importantly, these reactions lead to a time-dependent gelation that changes the ink rheology over time, and thus provides a simple basis to scan many ink rheologies and understand their effects on printability by direct ink writing. Little work has been done on using boehmite for DIW^10^, despite its common usage in extrusion processes^11^. In addition, there has been no specific focus so far on obtaining boehmite based dense materials.

The goals of this work are to develop a definition of printability for space filling, dense objects and link the printability of boehmite gels to their time-dependent rheological behaviour. Using parallel plate rheometry, we study the viscoelastic flow behaviour of boehmite gels as a function of time with special attention paid to the shear-dependent viscosity and yield stress. These measured rheological parameters are then compared to image analysis of printed objects to identify the physical forces behind shape deformation after printing. Slumping is usually ascribed to gravitational forces, supposing that the objects deform under their own weight if the yield stress after printing is not sufficiently high. But we show in this work that yielding can also be induced by capillary forces, that can even be stronger than gravity. Consequently, we develop a rheological criteria for printability based on dynamic yield stress and surface energy. Finally, we show that sintered objects printed from gels that meet this definition of printability are highly dense and mechanically resistant.

## Methods

### Materials

Raw powders for boehmite suspensions were obtained by mixing Catapal B (*Sasol*) with 2.5 *wt*% _AlOOH_ of nitric acid. Powders were seeded with 1.5*wt*% 30 nm (agglomerate size: 240 nm) *α*-Al_2_O_3_ particles (*S*_*BET*_ = 84 *m* ^2^.*g* ^−1^). Seeding allows a denser and finer microstructure at lower sintering temperatures (1300 °C)^12^. All suspension components (boehmite, acid, and seeds) were dispersed and drum dried to obtain the raw powder.

### Suspension preparation

Suspensions were prepared with deionized water. After adding half of the necessary amount of powder, suspensions were sonicated for 1 min and mixed under vacuum for 15 min in a Harnisch & Rieth D-VM-10 mixer. The previous steps were repeated after adding the rest of the powder. The boehmite mixture used for this study has been optimized for other ceramic processes with a 2.5% [HNO_3_]/[Al] ratio. Immediately after preparation, 43 wt% and 45 wt% suspensions are still liquid. Suspensions were poured into 30 cc plastic syringes (*EFD*, *Nordson*) and allowed to gel.

### Rheological properties and surface tension

For each suspension, rheological data were gathered at different aging times. *t* = *0* was defined as the moment when suspension preparation was finished. Rheological properties were determined with a Kinexus rotational rheometer (*Malvern*), using a 20 mm parallel plate geometry and a 20 mm pedestal plate. Both the geometry and the support were sandblasted to avoid slipping. A PEG heat-exchanger stabilized the temperature at 25 °C ± 0.05 °C. Elastic and viscous moduli (*G*′ and *G*′′) were obtained by amplitude sweep measurements at 1 Hz in the range of 0.1 − *σ*_*y*_ Pa. *G*′ and *G*′′ were taken at plateau values in the linear viscoelastic region (LVR).

**Figure 1:**
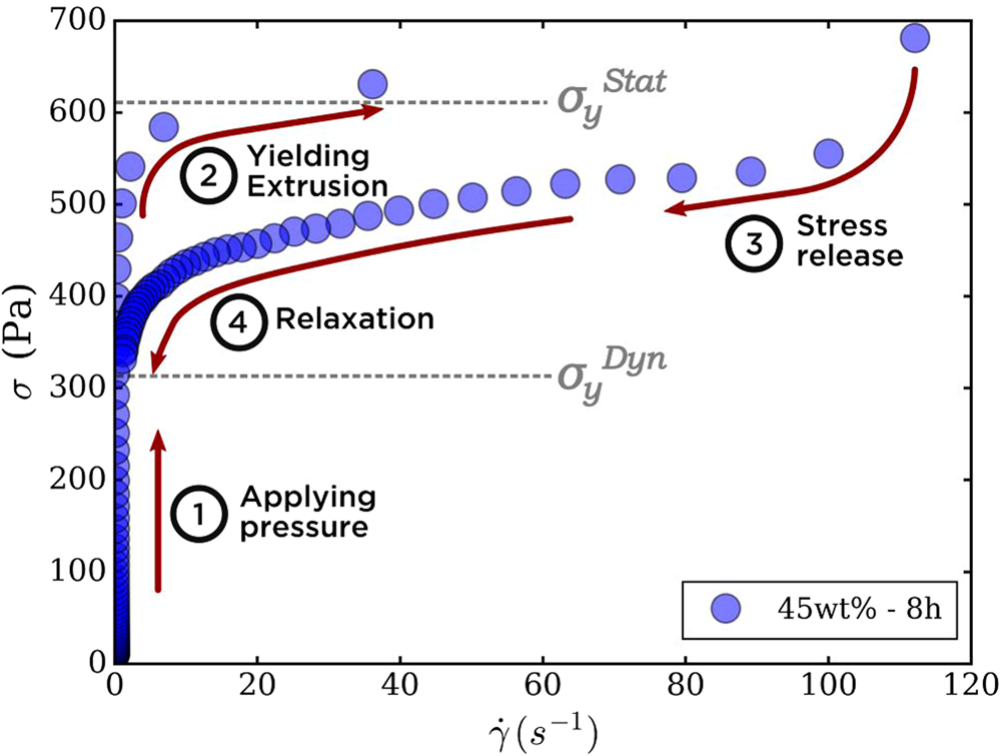
Loop flow curve of a 45 wt% boehmite suspension aged for 8 h. This protocol replicates the shear applied on a printed ink, from the syringe to the printing support. While measuring $\dot{\gamma }$, an increasing stress controlled ramp was applied (1) until suspension yields at the ${\sigma }_{y}^{Stat}$ (2). When $\dot{\gamma }$ exceeded 100 *s* ^−1^ (3), a decreasing shear rate controlled ramp was applied until $\dot{\gamma }=1\,{s}^{-1}$ and stress relaxes at ${\sigma }_{y}^{Dyn}$ value (4).

Static and dynamic yield stress (${\sigma }_{y}^{Stat}$ and ${\sigma }_{y}^{Dyn}$, respectively) were measured with a Stress-Shear rate loop as described in Fig. 1. This loop retraces the shear history of boehmite inks printed in a DIW device. A stress is applied (step 1) until suspension is extruded (step 2) through the nozzle at a certain shear rate (step 3). Stress relaxation leads to ${\sigma }_{y}^{Dyn}$ once printing is completed. The value of ${\sigma }_{y}^{Stat}$ was estimated at the slope change onset in the first part of the loop, and ${\sigma }_{y}^{Dyn}$ was obtained at the y-intercept after fitting the data to the Herschel-Bulkley equation.

The surface tension of 45 wt% suspension and deionized water was measured by pendant drop method, using an *ImageJ* plug-in ref. 13. Drops were suspended from 1.37 mm diameter nozzles and images were taken with a digital microscope with an intense backlight to increase contrast.

### Printing procedure and image analysis

Samples were printed by Direct Ink Writing (DIW), using a Nordson SL940 slurry deposition system. A plunger is air pressure-driven through the syringe to dispense the suspension while the printing head moves along programmed X-Y and Z axes. The operator manually adjusted the pressure to ensure adequate flow through the nozzle, as a function of printing speed. Each layer started by dispensing at the starting position for 500 ms (±100 ms, depending on the applied pressure), and finished with a short non-dispensing horizontal line to avoid a common defect generated by the vertical movement of the printing head.

At different aging times for both suspensions, lines were superimposed at 3 mm.s^−1^ to build an object 14 mm long, about 5 mm high, and nozzle size width object. The printing was filmed at 2 frames per second, against a black matt background and support to enhance contrast and decrease reflections in post-treatment. Collected images were thresholded, cropped, and binarized with a custom Python script. White pixels, corresponding to the printed object, were summed to obtain the area *A*. The theoretical area *A*_*th*_ was calculated as *A*_*th*_ = *lnd*, were *l* (mm) is the printed length, *d* the nozzle diameter (mm), and *n* the number of stacked layers. The ratio *A*/*A*_*th*_ was automatically calculated for each frame.

Three-point bending samples required additional precautions in printing paths. All tested samples were printed with 500 *μm* nozzles. To prevent printing defects induced by insufficient line merging, objects were printed alternatively in lengthwise and widthwise directions. Each line was programmed to overlap the adjacent one in each layer by 10% of the nozzle size (in this case, 500 *μm*). Bars were printed on microscope glass slides and dried for 2–3 days at room temperature and 90 rh% and then at ambient humidity until the sample became opaque. All samples were sintered at 1300 °C for 60 min and 5 °C/min ramp. Three-point bending tests were performed on 22 unpolished bars with a *Shimadzu AGSX* press at 0.2 mm.s^−1^. The span between bending points was 12 mm. Average sample size was 16.7 × 2.2 × 2.1 mm^3^.

## Results and Discussion

### Rheological Behaviour of Aging Boehmite gels

**Figure 2:**
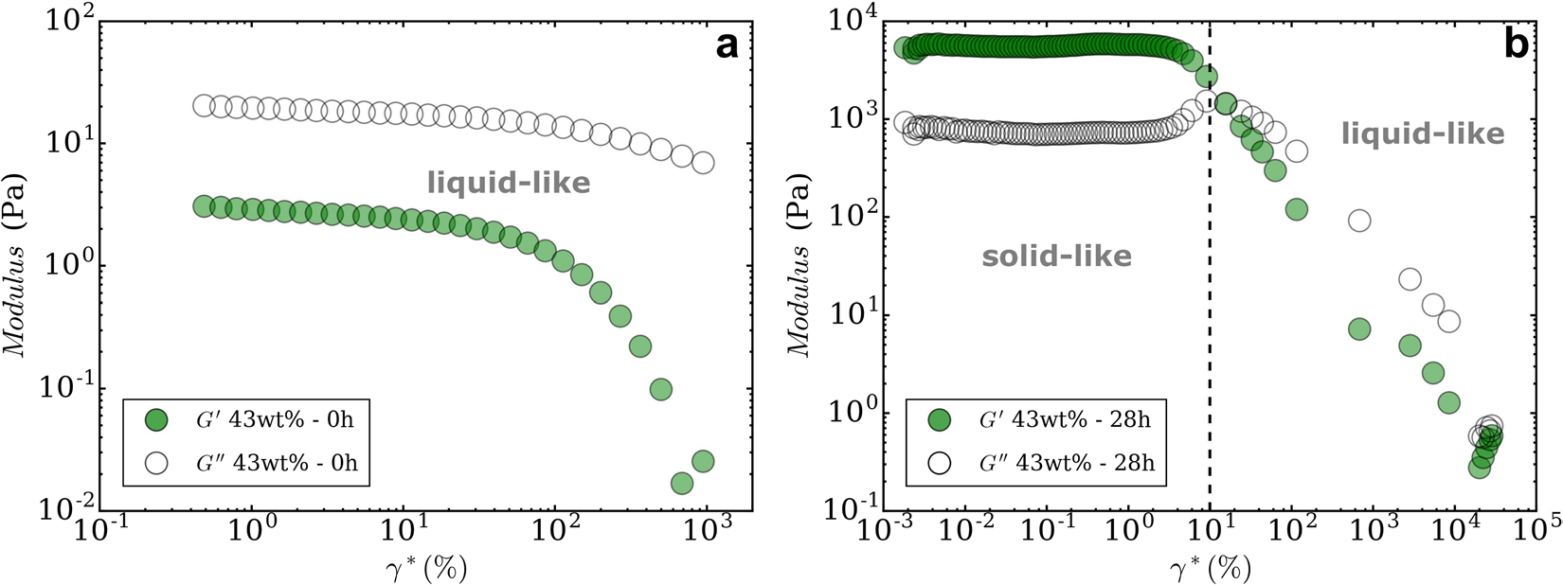
Elastic and viscous modulus vs. strain obtained by amplitude sweep at 1 Hz for 43 wt% boehmite suspension after aging for (**a**) 0 h and (**b**) 28 h. Before gelation, *G*′′ values are always higher than *G*′ at all strain values, unlike at 28 h where the gel transitions from a solid-like to a liquid-like behaviour.

Viscoelastic suspensions are characterized by their complex shear modulus:

1 $${G}^{\ast }=G^{\prime} +iG^{\prime\prime} $$

where *G* ^*^ is composed of a the real component (*G*′), the elastic (or storage) modulus, and an imaginary component (*G*′′), the viscous (or loss) modulus. Figure 2 shows *G*′ and *G*′′ vs *γ* ^*^(shear strain) ﻿in parallel plate oscillatory measurements at 1 Hz for 43 wt% solids loading boehmite gel aged for 0 h (a) and 28 h (b). These two examples are indicative of the general rheological behaviours observed in all suspensions. For the 0 h aged sample, *G*′′ is greater than *G*′ for all strains and the ink has a liquid-like behaviour. After aging for 28 h, *G*′ is greater than *G*′′ up to 10% strain, at which point *G*′′ increases and intersects *G*′. After this intersection, both moduli decrease with increasing *γ* ^*^ and *G*′′ is greater than *G*′. The behaviour of the gel aged for 28 h is emblematic of a viscoelastic response where the rheological behaviour at strains <10% is linearly viscoelastic and 10% is the yield strain, *γ*_*y*_.

**Figure 3:**
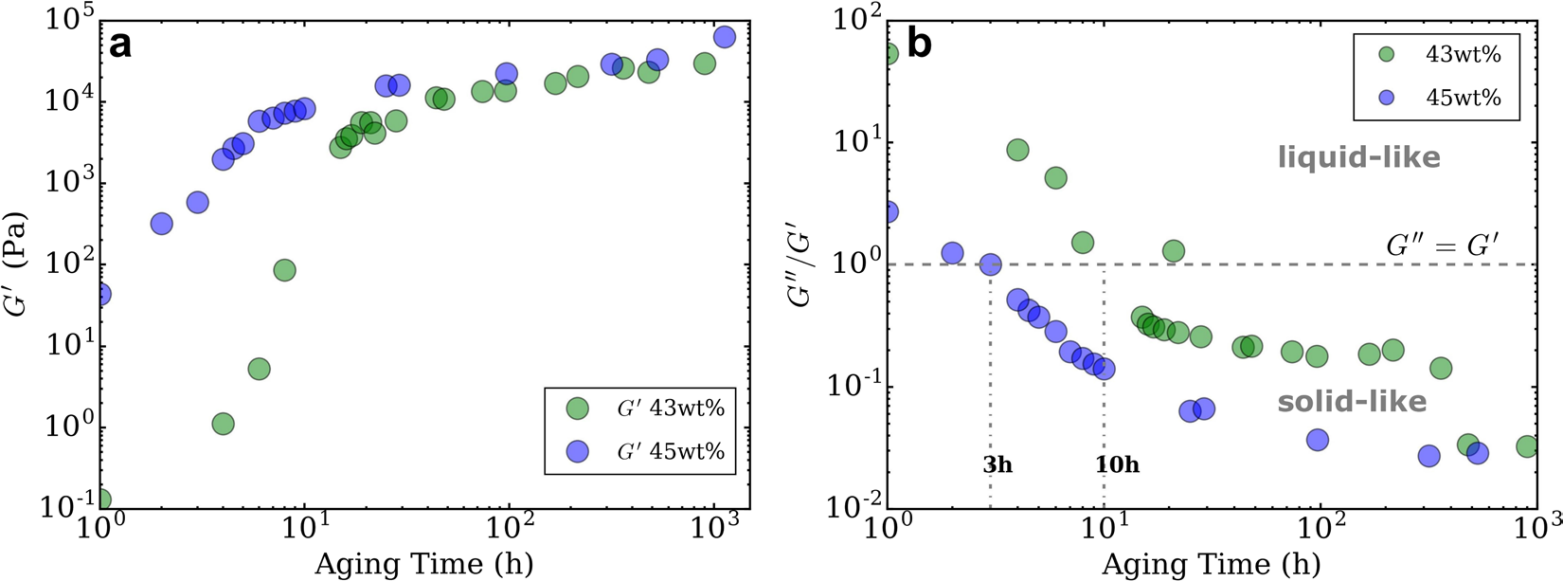
(**a**) Evolution of elastic modulus G′ for 43 and 45 wt% suspensions measured at 1 Hz (**b**) Evolution of *G*′′/*G*′ as a function of aging time for 43 and 45 wt% solids loading.

To assess the significant rheological changes in boehmite gels, samples were characterized over aging times from 0 to 1200 h. The evolution of elastic modulus *G*′ as a function of aging time for 43 and 45 wt% solids loading gels is shown in Fig. 3a.

Elastic modulus for both suspensions increases with aging time. For the 45 wt% gel, *G*′ starts at 40 Pa and increases over two orders of magnitude from 1 h to 10 h aging time. For the 43 wt% sample, the general behaviour is the same, but shifted towards later aging times. The elastic modulus is always lower than for the 45 wt% suspension. After 100 h, both moduli values are very close (≈10^4^–10^5^) compared to early aging times, where the 45 wt% *G*′ is higher by 2 orders of magnitude.

To provide more insight into the fluid-like versus gel-like behaviour, Fig. 3b shows the ratio *G*′′/*G*′ vs aging time. *G*′′/*G*′ is a useful quantifier of viscoelasticity, as the gelation point is defined by *G*′′/*G*′ = 1^14^. *G*′′/*G*′ values < 1 indicate a solid-like elastic dominant behaviour, while values > 1 signify a liquid-like viscous dominant behaviour. 45 wt% suspension reached gelation point after 3 h while it took 10 h for the 43 wt% sample. *G*′′/*G*′ for both samples decreases with aging time, becoming relatively steady after 10 h indicating relatively stable rheological behaviour. Importantly, the *G*′′/*G*′ values for the 43 wt% inks are generally higher than the 45 wt% inks, which indicates that the lower solids loading inks have a greater contribution of viscous, liquid-like behaviour in their overall rheology.

Ceramic suspensions with solids loadings *ϕ* ≥ 0.3 and G′ ≥ G′′ usually exhibit a shear-thinning flow behaviour, approximated by the Herschel-Bulkley model^14^:

2 $$\sigma ={\sigma }_{y}^{Dyn}+K{\dot{\gamma }}^{n}$$

where *σ* is the stress (Pa), ${\sigma }_{y}^{Dyn}$ is the dynamic yield stress (Pa), *K* is a model factor (consistency index in Pa.s^*n*^), $\dot{\gamma }$ is the shear rate (s^−1^), and *n* is the flow index.

**Figure 4:**
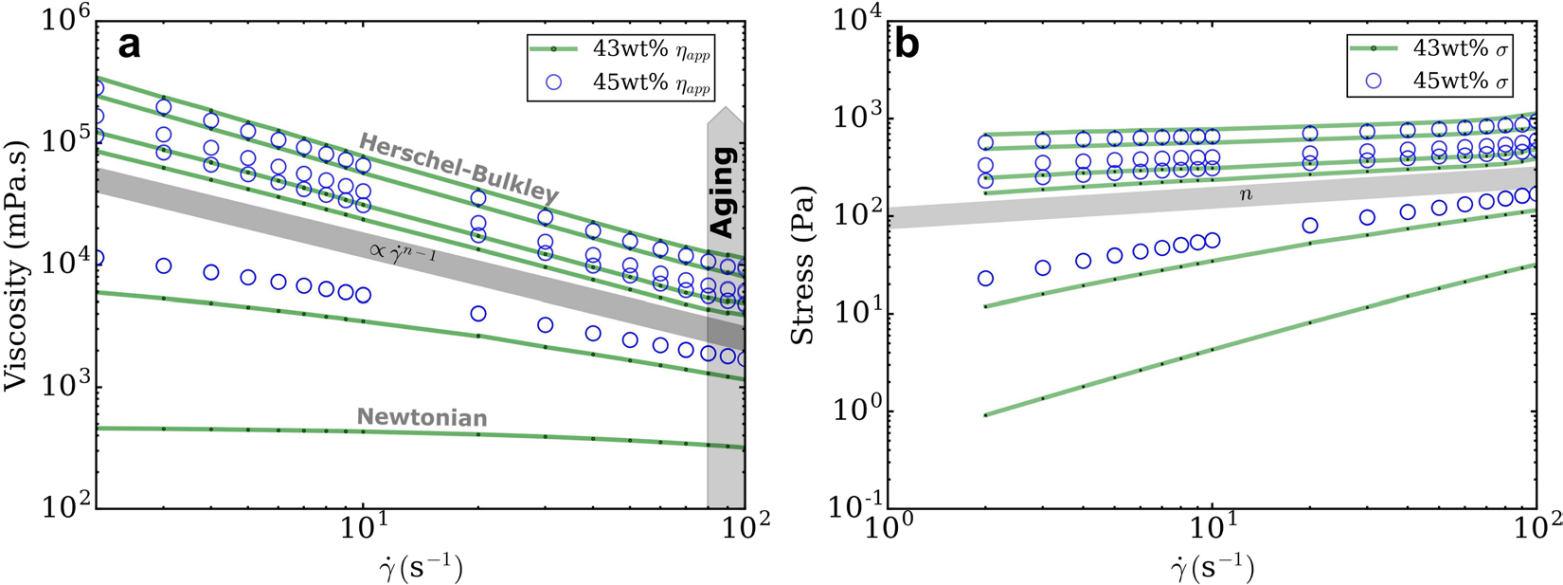
Evolution of viscosity *η*_*app*_ (**a**) and stress *σ* (**b**) as a function of shear rate $\dot{\gamma }$ for 43 and 45 wt% suspension at respectively *t*_*aging*_ = 0, 6, 16, 21, 44, 96 *h* and *t*_*aging*_ = 0, 5, 8, 25 *h*, from bottom to top for both plots.

Figure 4a shows a log-log plot of viscosity versus shear rate and Fig. 4b log-log plot of stress versus shear rate for 43 wt% (green lines) and 45 wt% (blue hollow circles) solids loading inks. In Fig. 4a, for the 43 wt% suspension, viscosity is independant from shear rate at *t*_*aging*_ = 0 *h*, indicating a Newtonian behaviour (slope = 0). On the contrary, 45 wt% exhibits a shear-thinning behaviour at *t*_*aging*_ = 0 *h*, with a shear-thinning index (proportional to the slope) lower than after gelation. Zero-shear viscosity values increase from 10^2^ to 10^5^ mPa.s with time. 43 wt% gel also progressively becomes a shear-thinning fluid, until gelation point, where a yield stress appears (Herschel-Bulkley model). After gelation, there is no increase in shear-thinning behaviour, since all slopes are equal and proportional to ${\dot{\gamma }}^{n-1}$ (with *n* roughly equal to 0.5) despite increasing viscosity. Compared to the 45 wt% (blue hollow circles), there is no effect of solids loading on the amount of shear-thinning, as the slope for 45 wt% gels is identical to 43 wt% gels. A similar behaviour is observed in Fig. 4b, showing stress as a function of shear rate. For 43 wt% gel, *log σ* increases with aging time and is linear with $log(\dot{\gamma })$ for samples beyond gelation point. As expected by the logarithmic linearization of the Herschel-Bulkley equation, the grey line represents the constant slope *n*, with no increase in shear-thinning effect after gelation.

In summary, both solids loadings inks show shear-thinning behaviour for aging times beyond the gelation time. However, for all aging times and shear rates, the viscosity of the 45 wt% inks is higher than the 43 wt% inks. This is consistent with the more elastic-like behaviour observed for the 45 wt% inks observed in Fig. 3b.

Yield stress is another important rheological parameter for viscoelastic fluids. For some fluids, two different types of yield stress can be defined: the static yield stress ${\sigma }_{y}^{Stat}$ which is the stress required to flow from a rest state, and the dynamic yield stress ${\sigma }_{y}^{Dyn}$ which is the minimum stress required for a fluid in motion to continue flowing. As the inks flow through the nozzle tip, it induces a shear rate that is maximal at the walls and can be estimated by^15^:

3 $${\dot{\gamma }}_{max}=\frac{4\dot{Q}}{\pi {r}^{3}}$$

with *r* the nozzle radius, and $\dot{Q}$ the volumic flow rate, calculated as $\dot{Q}=S{r}^{2}$, with *S* the printing speed. For example, *γ*_*max*_ = 61 *s* ^−1^ for a 500 *μm* diameter nozzle, but is doubled for 250 *μm* nozzles. Thus, a distinction between both yield stresses is important because the inks must flow through the nozzle, overcoming ${\sigma }_{y}^{Stat}$, but at the same time, enduring high shear rates and still form objects with minimum deformation after printing, which requires a sufficient ${\sigma }_{y}^{Dyn}$. Thus these values provide relevant process parameters for DIW including minimum stress applied to the inks to start flowing, minimum stress required for continuous flow and printing, and maximum stress that can be endured after printing without causing deformation.

**Figure 5:**
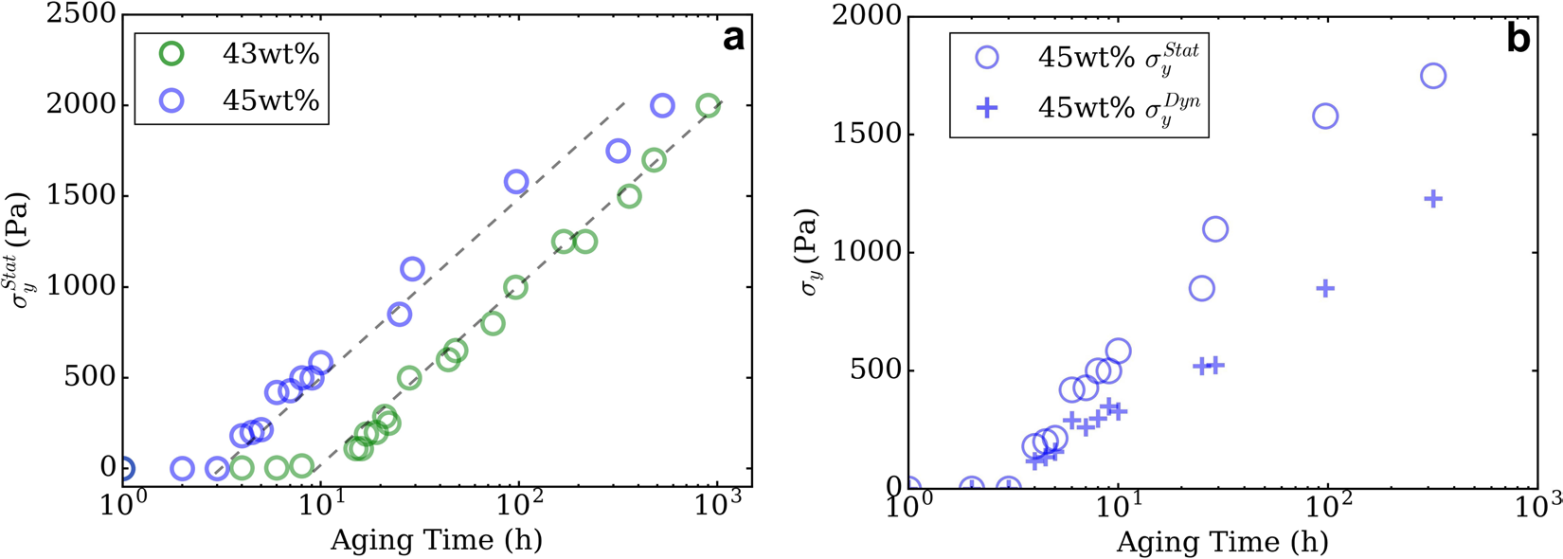
(**a**) Evolution of static yield stress ${\sigma }_{y}^{Stat}$ as a function of aging time for 43 wt% and 45 wt% boehmite suspensions (**b**) Static and dynamic yield stresses evolution as a function of aging time for 45 wt% suspension.

Figure 5a shows a semi-log plot of ${\sigma }_{y}^{Stat}$ and ${\sigma }_{y}^{Dyn}$ as a function of aging time for both solids loadings. Two regions can be distinguished: a region corresponding to aging times below gelation point and another one beyond gelation point. In the first region, 43 wt% inks are Newtonian fluids, as observed in Fig. 4a, and do not exhibit any yield stress. After gelation, ${\sigma }_{y}^{Stat}$ linearly increases with *logt*_*Aging*_ for both solids loading with comparable slopes. A similar behaviour is observed for ${\sigma }_{y}^{Dyn}$. It linearly increases with *logt*_*Aging*_, although the slope is different than for ${\sigma }_{y}^{Stat}$, as it is shown in Fig. 5b. In fact, for the 45 wt% suspension, between 3 h and 7 h, the difference between ${\sigma }_{y}^{Stat}$ and ${\sigma }_{y}^{Dyn}$ is small. After 8 h, they separate, and ${\sigma }_{y}^{Stat}$ is always greater than ${\sigma }_{y}^{Dyn}$ at all aging times. For both solids loadings, the divergence of the yield stresses occurs several hours after the gelation point. As explained in Fig. 4b, for a finite shear rate, stress values increase with aging time and solids loading. In accordance, Fig. 5a shows that at all aging times beyond the gelation point, ${\sigma }_{y}^{Stat}$ is higher for 45 wt% than for the 43 wt% suspension. The same observation was made for ${\sigma }_{y}^{Dyn}$, with lower values than ${\sigma }_{y}^{Stat}$. The boehmite chemical structure is typical of this difference between yield stresses as higher ${\sigma }_{y}^{Stat}$ values are required to break electrostatic bonds between boehmite clusters, but once at rest, unbroken chemical bonds between boehmite particles maintains a residual ${\sigma }_{y}^{Dyn}$.

Figure 5c,d also show that ${\sigma }_{y}^{Stat}$ and ${\sigma }_{y}^{Dyn}$ diverge after 20 h for 43 wt% suspension and after 6 h for 45 wt%. This separation supports the existence of two structures inducing boehmite gelation, specially since these aging times do not match with gelation point (Fig. 3b, *G*′′/*G*′ = 1). It also justifies the distinction between ${\sigma }_{y}^{Stat}$ and ${\sigma }_{y}^{Dyn}$ for DIW suspensions as the first must be outreached to extrude and the latter must be high enough to build an object.

### Printability

Clear rheological criteria exist for printing spanning, woodpile type structures^16, 17^, but there has been little systematic exploration of the printability of dense and strong structures that need to efficiently and completely fill space rather than spanning voids. Therefore, printability is not universally defined by the rheologies previously reported, it is also depends on the application. Even among the subset of DIW applications involving dense structures, defining printability is subjective and is frequently posed as an engineering tolerance problem: how much deformation can the structure tolerate before functionality is harmed? It is also important to identify the physical parameters behind shape deformation. How much slumping is induced by gravity and are capillary forces equally important to consider?

#### Quantifying deformation

**Figure 6:**
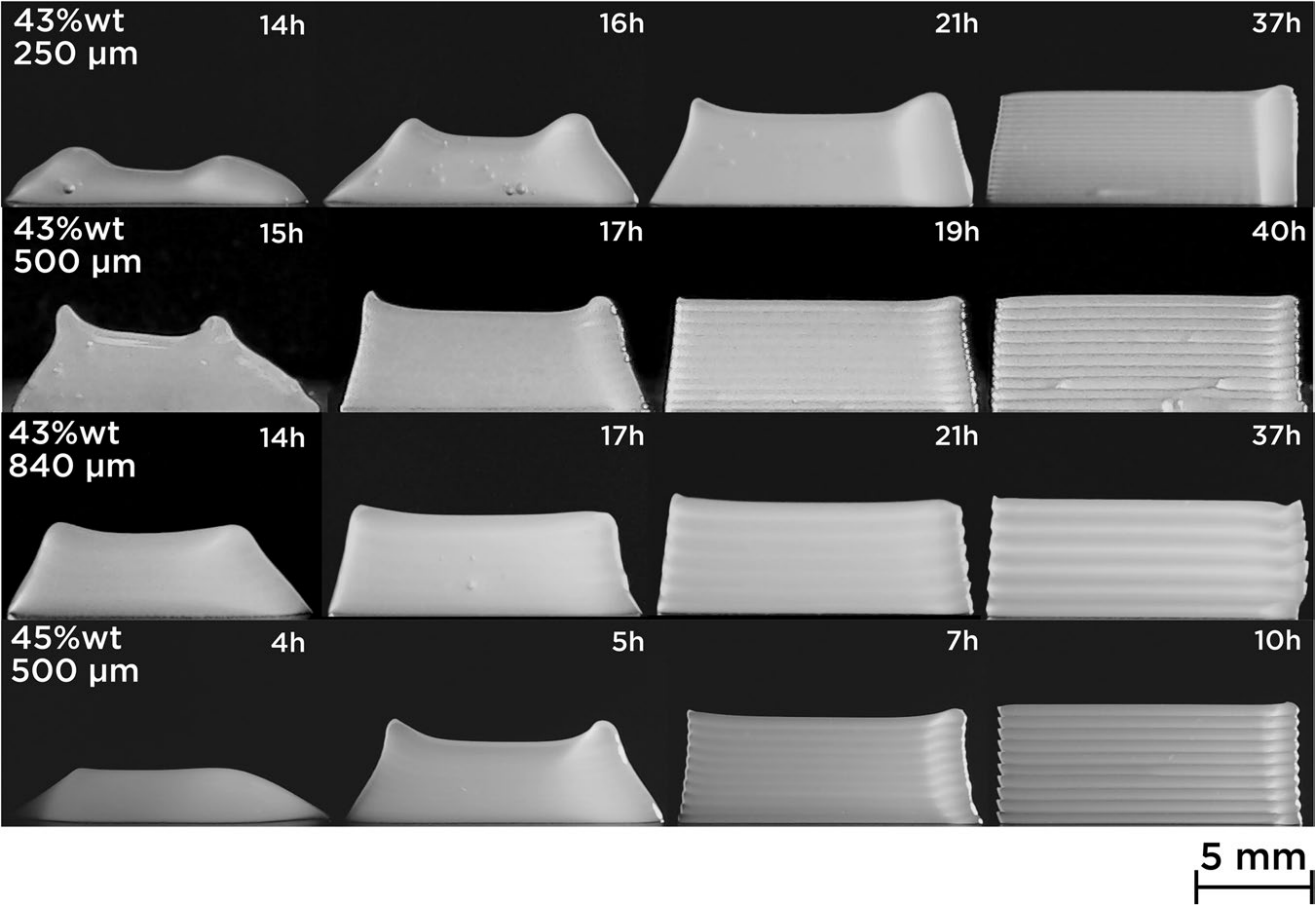
5 mm stacks of 43 wt% and 45 wt% boehmite suspensions printed with 250, 500 and 840 *μm* nozzles at 3 *mm*.*s* ^−1^ after different aging times. 45 wt% suspension became printable earlier than 43 wt%, whatever than nozzle size is. Nozzle size also impacts the printability as comparable aging times lead to different results with the same suspension. With a ${\sigma }_{y}^{Dyn}$ of 200 Pa, 5 h-45 wt% object clearly confirms that yield stress is not a sufficient criterion.

To develop a definition of printability, we will characterize the areal deformation of DIW printed objects as a function of ink rheology. Figure 6 shows several objects printed with different solids loadings at different aging times. Single lines were stacked to print a 14 × 5 *mm* object (from the right to left of each object on the images), and width was equal to nozzle diameter. At low aging times (first column), all samples exhibit high deformation. 43 wt% objects printed 840 *μm* nozzle better support layer stacking, as the deformation is lower than for 250 *μm* and 500 *μm* nozzles, at equivalent rheological properties. This is confirmed at around 17 h and 20 h. Earlier pictures of objects printed with 43 wt% suspensions are not presented because they invariably produced sessile droplets, and thus, where not relevant in measuring areal deformation. With 45 wt% ink and 500 *μm*, the obtained object is very close to a droplet, but a certain form of shaping can already be observed. With increasing aging time, deformation progressively decreases in all conditions. After 21 h, 43 wt% suspension printed with 250 *μm* nozzle slumped more than with 500 and 840 *μm*. 45 wt% inks provided the same result after aging for only 7 h. This result is consistent with the difference in gelation kinetics with solids loading described in the rheological characterization (Figs 3b and 5). Objects printed after 10 h with 45 wt% suspension did not show any deformation which is not the case with the same nozzle and lower solids loading after about twice the time (19 h). The visibility of the printed lines is also a good indicator of slumping or excessive line merging. Lines were not visible with the 250 *μm* nozzle until late aging times (37 h) while they could be clearly discerned with larger nozzles at equivalent aging time. Also for the 250 *μm* nozzle, slumping occurred asymmetrically, as observed after 16 and 21 h. This can be explained by longer dispensing times at starting points to avoid initial printing defects, hence locally increasing the objects’ width.

#### Gravitational slumping

If we take a closer look at 45 wt% inks printed after 5 h, they still led to slumped objects, despite exceeding gelation point (Fig. 3b) and 200 Pa ${\sigma }_{y}^{Dyn}$ (Fig. 5). Considering a ${\sigma }_{y}^{Dyn}$ of 200 Pa, an estimation of the maximum printable height *h*_*max*_ can be calculated:

4 $$\begin{array}{ccc}{h}_{max} & = & \frac{{\sigma }_{y}^{Dyn}}{\rho g}\\ {h}_{max} & \approx  & 1.5\,cm\end{array}$$

with *ρ* = 1.40 *kg*.*m* ^−3^ and 1.43 *kg*.*m* ^−3^, respectively for 43 wt% and 45 wt%; *g* = 9.81 *m*.*s* ^−2^. With a 500 *μm* nozzle, that corresponds to approximately 30 layers. But Fig. 6 shows the slumping at 5 h induced by stacking only 10 layers (5 *mm*) of a 45 wt% boehmite suspension, despite a theoretically sufficient yield stress. Even though slumping decrease is correlated with separation in ${\sigma }_{y}^{Stat}$ and ${\sigma }_{y}^{Dyn}$ values reported in Fig. 5c,d (*t*_*sep*_ = 20 h for 43 wt% and *t*_*sep*_ = 6 h for 45 wt%), printability can not be defined by the yield stresses alone.

#### Impact of capillary forces

**Figure 7:**
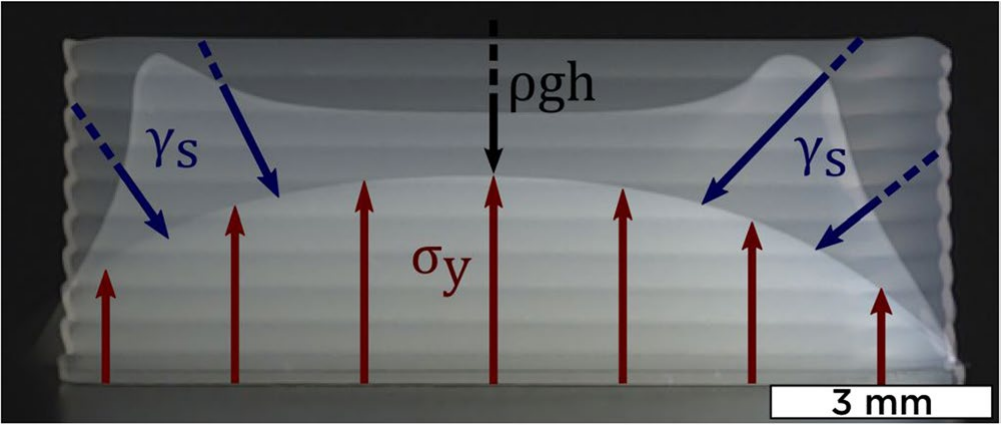
Frame superposition of 45 wt% boehmite gel printed after 1 h, 5 h, and 10 h of aging, with a 500 *μm* nozzle.

Figure 7 shows a superposition of three different frames taken from objects printed at separate aging times with 45 wt% suspension: 1 h, 5 h and 10 h. After aging for 1 h, 45 wt% suspension still behaves as a Newtonian fluid with no yield stress (Fig. 5). The printed lines turn very quickly into drops as surface tension forces reduce overall interface energy. At 5 h, middle shape shows that 45 wt% inks start to support stacking, with a characteristic double peak shape (corresponding to printing start and end). The object is not only slumping because of gravitational forces (black arrow), but surface tension *γ*_*s*_ (blue arrows) tends to bring the extremities towards the center to reduce surface energy and turn into a drop, until stabilizing into the intermediate red shape. Finally, the height and length of the top shape both correspond to the path programmed in the printer. There are no signs of slumping and each printed layer is clearly discernible. Hence, after aging for 10 h, 45 wt% suspension is considered to be printable. Compared to most commonly printed colloidal suspensions, boehmite gels have a significantly lower solids loading, and surface tension forces contribute more to deformation in the few seconds following printing. Studies usually insist on the importance of yield stress to prevent gravitational slumping^4, 18, 19^, but very few reported surface tension’s relevance in DIW suspensions^20, 21^, as it is considered to be more important for droplet based techniques^17, 19^.

#### Combining gravity and capillarity

In the light of these arguments, we suppose that ${\sigma }_{y}^{Dyn}$ should support printed object’s own weight, but also capillary forces tending to minimize surface energy, such as a suspension becomes printable when forces are balanced. Since we proved in Eq. 4 that gravity is not sufficient, we evaluate the relative impact of both suspected physical forces using two dimensionless numbers Ξ (Xi) and Ξ_*γ*_ such as:

5 $${\rm{\Xi }}=\frac{{\sigma }_{y}^{Dyn}}{\gamma {R}^{-1}+\rho gh}\quad and\quad {{\rm{\Xi }}}_{\gamma }=\frac{{\sigma }_{y}^{Dyn}}{\gamma {R}^{-1}}$$

with *R* the nozzle diameter and *γ*_*s*_ equal to the suspension’s surface tension. Measuring the surface tension of a yield stress fluid is not trivial and extremely influenced by the rheological properties and protocol^22^. Measures were performed on five different drops and the means values were very similar (about 1.3% difference in surface tension between boehmite and water). This in accordance with other studies^23^ showing that the surface tension of a yield stress fluid corresponds to the surface tension of its solvent medium (in this case water, 72 *mN*.^−1^).

**Figure 8:**
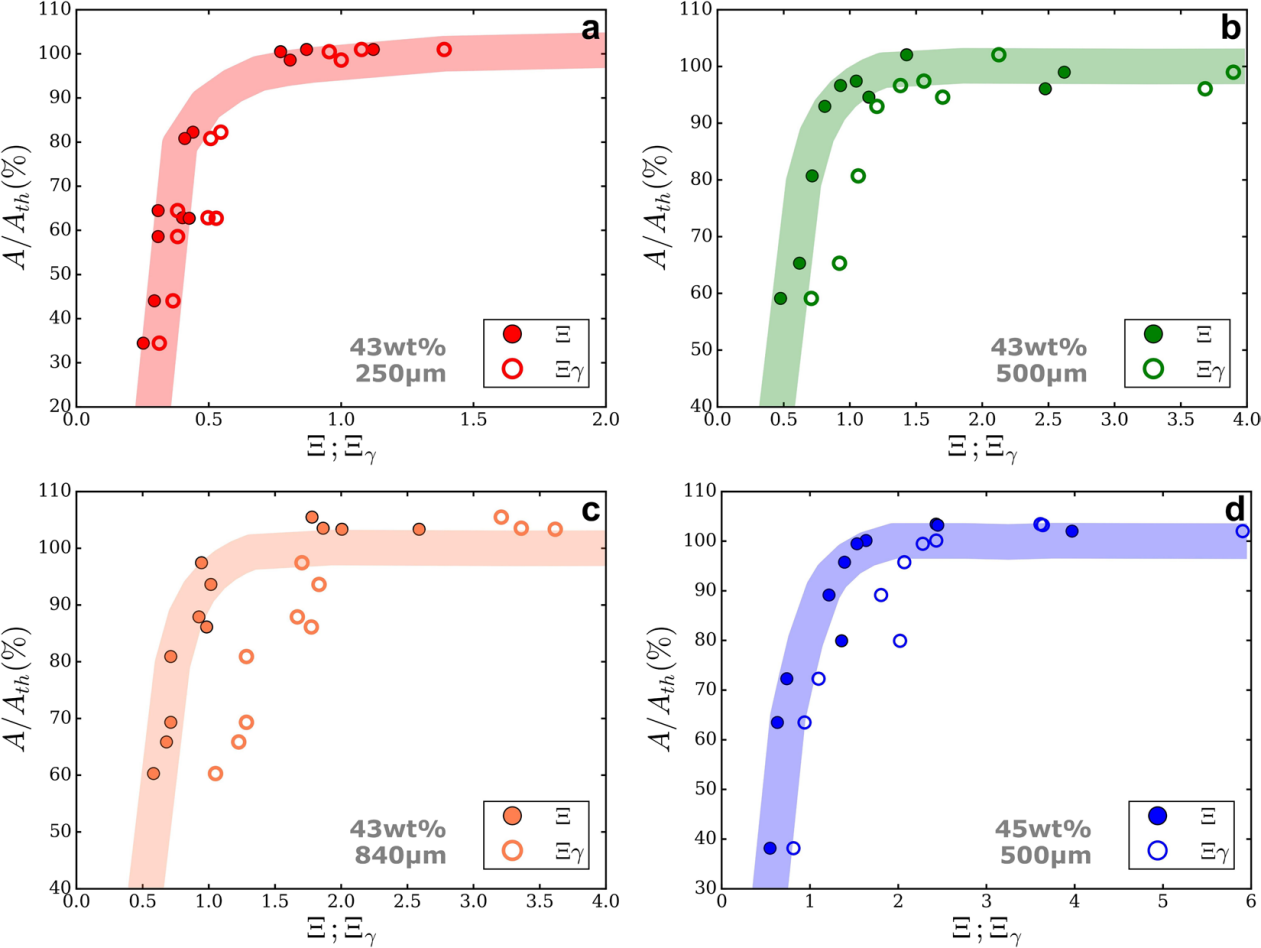
Deformation ratio vs Ξ and Ξ_*γ*_ for 43 wt% boehmite suspension with (**a**) 250 *μm* (**b**) 500 *μm* (**c**) 840 *μm* nozzle and (**d**) 45 wt% with 500 *μm*. Rheological measures for the 43 wt% suspension come from different suspension to ensure an exact correspondance between dynamic yield stress and deformation ratio. Colored stripes were plotted to help see the trend.

**Figure 9:**
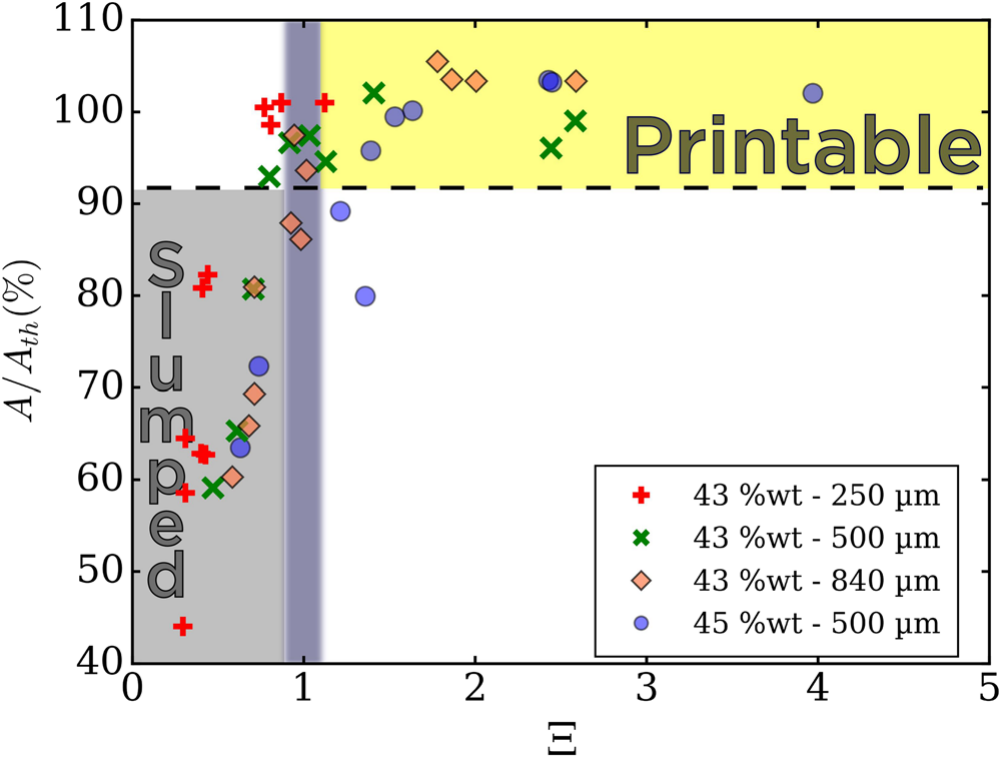
*A*/*A*_*th*_ vs. Ξ of both boehmite suspensions and different nozzles sizes. Ξ = 1 establishes a clear limit of printability as all the points beyond have a shape fidelity higher than 90% (Printable region, in yellow). For Ξ < 1, the obtained objects are deformed and suspension is considered as non printable (Slumped region, grey).

By performing image analysis at several aging times on frames similar to those in Fig. 6, *A*/*A*_*th*_ can be defined as an objective deformation index. *A*/*A*_*th*_ is the ratio between the printed object’s area *A* in each frame and the theoretical area *A*_*th*_ programmed with the printer. Figure 8 shows the evolution of *A*/*A*_*th*_ as a function of Ξ and Ξ_*γ*_, calculated with all the rheological data gathered throughout suspension’s gelation time. Each point in the plot corresponds to the image analysis result of an object printed after a certain aging time and its corresponding value for Ξ and Ξ_*γ*_. Since all printed samples have approximately the same height, the term *ρgh* is constant. To change the capillary term, we decided to use different nozzle sizes. In fact, we did not succeed in significantly changing the boehmite gels’ surface tension without changing essential parameters for boehmite peptization. *A*/*A*_*th*_ evolution is similar for both dimensionless numbers, solids loadings, and nozzle sizes. It sharply increases from 40–60% for both dimensionless numbers values between 0 and 1, and reaches a plateau of 100% for higher Ξ and Ξ_*γ*_ values. Between Ξ_*γ*_ and Ξ, values are shifted to the left. This shift is more important as the nozzle size increase, as it can be observed for the 840 *μm* nozzle (Fig. 9c). Ξ includes the effect of gravitational slumping, but Ξ_*γ*_ does not. Logically, gravity plays a more substantial role when nozzle size increases because more material is being deposited. In fact, the shift is almost negligible in Fig. 9a with the 250 *μm* nozzle. Even if summing capillary and gravitational forces is not trivial, it seems that Ξ provides a more conclusive criterion for printability. Ξ = 1 corresponds indeed to *A*/*A*_*th*_ values around 90%.

In Fig. 9, the blue line corresponds to Ξ = 1. All points fall approximately in two distinct regions. The grey region, where *A*/*A*_*th*_ is lower than 90% for Ξ inferior to one, and the yellow region, where *A*/*A*_*th*_ is higher than 90% and Ξ superior to one. Hence, grey region corresponds to non-printable suspensions and yellow region to printable suspensions, separated by Ξ = 1 that establishes a clear limit. As ${\sigma }_{y}^{Dyn}$ is getting closer to the sum of gravitational and capillary forces, printability increases. Thus, when inks’ yield stress after printing (${\sigma }_{y}^{Dyn}$) is able to overcome surface tension and gravity, objects can be printed without deformation. These results show that Ξ is a reliable dimensionless number in assessing printability for DIW inks.

### Microstructure and Flexural Strengths of *α*-Al_2_O_3_ Bars Obtained by DIW

**Figure 10:**
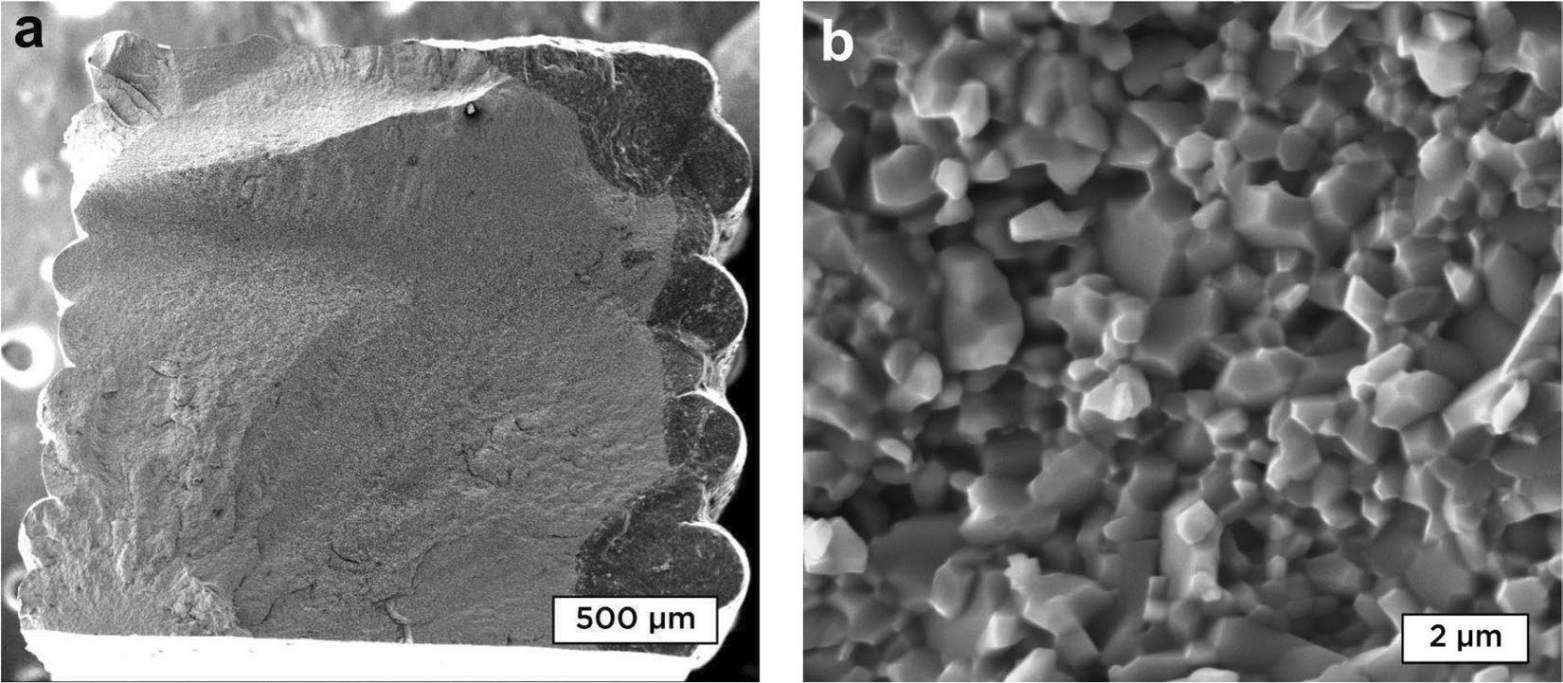
(**a**) SEM image at x120 of fracture surface from Al_2_O_3_ bar obtained by sintering boehmite printed with DIW. Fracture resulted from Three-point bending test. Stacked lines are not discernible in the center of the sample, but clearly visible on the samples surface. (**b**) SEM image at x10000 of the same fracture surface. The object is dense with a fine grained microstructure, suitable for high strength mechanical properties

When the rheological properties have been tailored in accordance to what was exposed in the previous section, the challenge for DIW is to produce dense space-filling ceramic objects with no printing defects. Figure 10a illustrates a fracture surface from an *α*-Al_2_O_3_ bar obtained by DIW. Boehmite bars have initially been printed, properly dried, and sintered to obtain bars for three-point bending tests. To avoid microdefects caused by bubbles formation, two drops of octan-1-ol (*Sigma-Aldrich*) per 100 mL of water were added as a defoaming agent to the boehmite suspensions used to print these samples.

#### Microstructure

Images show that there are no visible printing defects in the core of the sample. The sample surface is embossed by the printing path as it can be seen on the sides of the image. Because of the alternate printing directionality, some layers are more protuberant than the others, such as it is possible to distinguish each printed layer. These surface defects are inherent to most of additive manufacturing techniques as layer-by-layer building forcibly leaves traces on the sample boundaries. Figure 10 shows that on the contrary, inside the sample, all signs of layer stacking disappears. Lines have perfectly merged to each other to produce a dense object, and even after sintering, printing does not leave traces inside the sample and layers can not be distinguished. Figure 10b shows a higher magnification (x10k) of the same sample. The microstructure has an average grain size of 1 *μm*, with a high population of submicronic grains. Residual porosity is predominantly located at triple-point.

#### Density and flexural strength

Sample density reaches 97% of theoretical Al_2_O_3_ density. Three-point bending results on 22 unpolished samples showed an average flexural strength of 591 MPa (±149), with a Weibull modulus of 4.5. Strength values range from 281 MPa up to 858 MPa. Even if these values still present a moderate reliability and significant standard deviations, the mean value is higher than what is reported for colloidal alumina and also higher than commercially available material. Maximum values are also in the range of what was reported for HIP sintered transparent *α*-Al_2_O_3_.

## Conclusion

The time-dependent rheology of boehmite gels was linked to deformation of 3D printed objects in order to produce a dimensionless criterion for printability, Ξ. We showed that capillary forces are an important parameter in tailoring the rheological properties of DIW suspensions, and that deformation is not only caused by gravitational slumping. We expect Ξ to be an accurate predictor of printability for other DIW inks because it links physical parameters that are not related with the chemical properties of the inks. Thus, while it is not evident that all starting materials can result in inks with the rheological properties necessary for printability, those with a Ξ near one should be capable of producing low deformation, high density and high strength materials like those demonstrated here.

In the future, an upper limit for Ξ should be established where the capillary forces between the printed lines are not sufficiently strong to merge them into defect free, space-filling structures. A second, equipment based, upper limit should also be established where the pressure applied by the printing system is insufficient to extrude the ink through the chosen nozzle size. However, as described here, scientists can use Ξ to rationally design inks for printing dense and strong materials by tailoring their rheological properties such that Ξ ≈ 1.

### Data Availability Statement

The datasets generated during and/or analysed during the current study are available from the corresponding author on reasonable request.
